# HER2-low and HER2-zero in breast cancer between prognosis, prediction and entity

**DOI:** 10.18632/oncotarget.28598

**Published:** 2024-06-20

**Authors:** Marcus Schmidt, Hans-Anton Lehr, Katrin Almstedt

**Keywords:** breast cancer, HER2, HER2-low, prognostic, predictive

HER2 is a well-established prognostic and predictive factor in breast cancer, which is associated with a poor prognosis but also offers the chance of improved survival when treated with targeted therapies based on the monoclonal antibody trastuzumab [1], both in advanced (hazard ratio (HR) 0.82, 95% confidence interval (CI) 0.71 to 0.94, *P* = 0.004) and in early (HR 0.66, 95% CI 0.57 to 0.77, *P* < 0.00001) stages [2, 3]. The American Society of Clinical Oncology (ASCO)/College of American Pathologists (CAP) defines HER2-positivity as either 3+ by immunohistochemistry (IHC) or 2+ with amplification by *in situ* hybridization (ISH) [4]. Yet, the vast majority of breast tumors are considered HER2-negative (IHC 0 or 1+ or 2+ without amplification) by these criteria, and it has until recently been accepted that HER2-negative tumors do not benefit from trastuzumab-based therapy [5].

Now, results of randomized trials with trastuzumab-based antibody-drug conjugates (ADCs) such as trastuzumab deruxtecan (T-DXd) have fundamentally challenged this long-held view. They found that not only outright HER2-positive tumors, but also advanced breast cancers with low HER2 expression (1+ or 2+ ISH-negative) respond to T-DXd [6, 7]. Interestingly, T-DXd was investigated in a randomized phase 2 study (DAISY) not only in advanced HER2-positive and HER2-low breast carcinomas, but also in carcinomas without any HER2 expression [8]. The confirmed objective response rate (ORR) was positively associated with HER2 expression: HER2-positive 70.6%, HER2-low 37.5%, HER2-zero 29.7%. The authors concluded that although HER2 expression is a decisive factor for the efficacy of T-DXd, other mechanisms may also play a role.

Beside the role of HER2 as a predictive factor for treatment with trastuzumab or T-DXd, its prognostic impact has also been reevaluated. The prognostic and predictive significance of HER2-low and HER2-zero was investigated by Denkert and colleagues in 2310 patients with HER2-non-amplified primary breast cancers who were treated with neoadjuvant cytotoxic chemotherapy [9]. They showed that HER2-low was significantly more common in hormone receptor(HR)-positive than in HR-negative tumors (64.0% vs. 36.7%, *P* < 0.0001) and that HER2-low tumors had a significantly lower rate of pathological complete response (pCR) compared to HER2-zero in HR-positive tumors (17.5 vs. 23.6%, *P* = 0.024). No such difference was found in HR-positive breast cancers. The 3-year overall survival (OS) in HER2-low tumors compared to HER2-zero tumors was 91.6% vs. 85.8%, *P* = 0.0016. Interestingly, the OS of HER2-low tumors was significantly better only in HR-negative tumors (90.2% vs. 84.3%, *P* = 0.016), but not in HER2-positive breast cancers. Based on these results, the authors proposed HER2-low as a new subgroup of breast cancers. Obviously, the reproducible classification as HER2-low has an important predictive effect for ADCs such as T-DXd.

The detection of a prognostic impact of a HER2-low status prompted us to test the prognostic significance of HER2-low and HER2-zero in a historic cohort of 410 consecutive node-negative breast cancer patients who had not received any adjuvant systemic therapy, with a median follow-up of more than 15 years [10]. The majority of HER2-negative patients were classified as HER2-low (56.4%). In this untreated population, HER2-low patients had significantly longer disease-free survival (DFS) (67.5% vs. 47.3%, *P* < 0.001) and OS (75.4% vs. 66.8%, *P* = 0.009) than HER2-zero patients. The results of the multivariable analysis confirmed the independent prognostic significance of HER2 status (DFS: HR 0.556, 95% CI 0.409–0.755, *P* < 0.001; OS: HR 0.664, 95% CI 0.467–0.945, *P* = 0.023). In agreement with Denkert and colleagues, our results suggest that hitherto HER2-negative patients should be differentiated in HER2-low and HER2-zero.

However, the distinct prognostic significance and the proposed description of HER2-low as a new entity have not gone unchallenged by other groups. For instance, Pfeiffer and coworkers reported a large retrospective cohort study on 1136.016 breast cancer patients using the National Cancer Database [11]. In the total population, HER2-low tumors had a lower pCR than HER2-zero tumors (OR 0.89, 95% CI 0.86–0.92, *P* < 0.001)). HER2-low tumors had only a slightly better OS (HR 0.98, 95% CI 0.97–0.99, *P* < 0.001) than HER2-zero tumors. The authors concluded that these results do not support the classification of HER2-low breast cancer as a unique disease entity. Also, based on reads from a large prospective cohort study that included 5,235 early-stage breast cancer cases, Tarantino and colleagues argued against HER2-low breast cancer as a distinct biological subtype [12]. They reported a significantly higher pCR to cytotoxic chemotherapy in HER2-zero tumors compared to HER2-low (OR 1.84, 95% CI 1.27–2.70, *P* = 0.002), but, when the multivariable analyses were adjusted for confounding factors such as HR status, neither pCR nor survival retained their independent significance.

Several recently published systematic reviews and meta-analyses investigated the prognostic significance of HER2-low compared to HER2-zero in early breast cancer (Table 1) [13–17]. A HER2-low status was associated with a better OS in all but one meta-analysis. That analysis found no significant association between HER2-low and DFS in the overall population but only in the HR-positive subgroup (HR 0.96, 95% CI 0.94–0.99, *P* = 0.003) [17]. In fact, others have confirmed that the association of a HER2-low status with survival is strongest in HR-positive carcinomas [15]. Based on these results, Molinelli and coworkers concluded that HER2-low breast cancer cannot be considered a new biological entity and that its different prognostic characteristics are likely due to HR status [15].

**Table 1:** Systematic reviews and metaanalysis of the prognostic impact of HER2-low vs. HER2-zero in early breast cancer

Author	Studies (*N*)	Patients (*N*)	DFS HR (95% CI)	OS HR (95% CI)
Ergun et al., 2023 [13]	23	636,535	0.87 (0.83–0.92)	0.82 (0.74–0.91)
Tang et al., 2023 [17]	26	677,248	0.97 (0.92–1.02)	0.90 (0.85–0.97)
Li et al., 2023 [14]	18	93,317	0.82 (0.73–0.93)	0.87 (0.81–0.93)
Petrelli et al., 2023 [16]	25	34,965 (HER2-low)	0.89 (0.84–0.94	0.83 (0.76–0.9)
Molinelli et al., 2023 [15]	42	1797,175	0.86 (0.79–0.92)	0.90 (0.85–0.95)

Abbreviations: CI: confidence interval; DFS: disease-free survival; HR: hazard ratio; *N*: number; OS: overall survival.

In a most recently published prospective cohort study that was not yet included in the systematic reviews and meta-analyses described above, HER2-low had a positive impact on survival also in HR-negative patients (HR 0.54, 95% CI 0.33–0.91, *P* = 0.02) irrespective of other key covariates (HR 0.48, 95% CI 0.27–0.83, *P* = 0.009) [18]. The authors concluded that these findings raised the possibility that HER2-low breast cancer may be a unique entity.

Could it be that the problems with the HER2-low status might reside in its low diagnostic reproducibility? Fernandez and coworkers found only 26% concordance between 0 and 1+, compared to 58% concordance between 2+ and 3+ [19]. A recent update to the ASCO/CAP guidelines for HER2 testing in breast cancer noted that the distinction between IHC 0 and 1+ is now clinically relevant, but that it is premature to create new outcome categories for HER2 expression (e.g., HER2-low, HER2-ultra-low) [20].

In summary, we found an independent positive prognostic effect of HER2-low compared to HER2-zero in early breast cancer. This result has been confirmed in several other studies and at the meta-analysis level. However, based on the currently available study results, it can not yet be conclusively determined whether HER2-low can be considered a separate diagnostic entity. However, the fact that early randomized trials find that HER2-zero tumors may also benefit from trastuzumab-deruxtecan, the question of a distinct HER2-low entity may soon become obsolete.
